# Nitric oxide mediates activity-dependent change to synaptic excitation during a critical period in *Drosophila*

**DOI:** 10.1038/s41598-021-99868-8

**Published:** 2021-10-13

**Authors:** Carlo N. G. Giachello, Yuen Ngan Fan, Matthias Landgraf, Richard A. Baines

**Affiliations:** 1 Division of Neuroscience and Experimental Psychology, School of Biological Sciences, Faculty of Biology, Medicine and Health, University of Manchester, Manchester Academic Health Science Centre, Manchester, M13 9PL UK; 2 Department of Zoology, University of Cambridge, Cambridge, CB2 3EJ UK

**Keywords:** Development of the nervous system, Molecular neuroscience, Neural circuits, Synaptic plasticity, Developmental biology, Neuroscience

## Abstract

The emergence of coordinated network function during nervous system development is often associated with critical periods. These phases are sensitive to activity perturbations during, but not outside, of the critical period, that can lead to permanently altered network function for reasons that are not well understood. In particular, the mechanisms that transduce neuronal activity to regulating changes in neuronal physiology or structure are not known. Here, we take advantage of a recently identified invertebrate model for studying critical periods, the *Drosophila* larval locomotor system. Manipulation of neuronal activity during this critical period is sufficient to increase synaptic excitation and to permanently leave the locomotor network prone to induced seizures. Using genetics and pharmacological manipulations, we identify nitric oxide (NO)-signaling as a key mediator of activity. Transiently increasing or decreasing NO-signaling during the critical period mimics the effects of activity manipulations, causing the same lasting changes in synaptic transmission and susceptibility to seizure induction. Moreover, the effects of increased activity on the developing network are suppressed by concomitant reduction in NO-signaling and enhanced by additional NO-signaling. These data identify NO signaling as a downstream effector, providing new mechanistic insight into how activity during a critical period tunes a developing network.

## Introduction

As networks develop, sets of neurons become spontaneously active. Initial activity shows little coordination across the network. However, as circuits mature, particularly as sensory afferents become functional, there is a shift towards increasingly coordinated patterned activity^1,2^. This transition is often also a critical period: a developmental phase of heightened or altered plasticity during which activity exerts maximal influence to network properties^3^. It is notable that errors made as a direct result of perturbations during a critical period can become ‘locked-in’, such that subsequently the network is unable to correct those mistakes; while the same perturbations applied outside this period do not have this effect. A well described example are transient activity manipulations of visual inputs in developing mammals, which have permanent and maladaptive effects to the formation of ocular dominance, when targeted to the critical period of the visual system^4^.

Though critical periods have commonly been associated with mammalian sensory and cortical networks, we identified a critical period in the development in the locomotor circuit of an insect, namely the *Drosophila*, indicative that critical periods are probably a universal phenomenon of network development. Manipulating activity during the *Drosophila* critical period, a 2-h window in late embryogenesis (17–19 h after egg laying, AEL), is sufficient to permanently alter the developmental trajectory of the locomotor network. This manifests in lasting network changes that mimic those of well characterized seizure mutants. Specifically, we find lasting changes to synaptic transmission, network activity and prolonged seizure duration following an electric shock (a stressor) at the final larval stage, 5 days after the transient critical period manipulation^5^. As might be expected, the critical period coincides with the emergence of coordinated locomotor behavior in the developing embryo^6–8^. Our previous work demonstrated that the overall level of network activity during the critical period is instructive for appropriate adjustment. For example, a gain-of function mutation in a voltage-gated Na^+^ channel (*para*^*bss*^) leads to overall increased activity levels and makes animals seizure prone. However, transient optogenetic reduction of activity, during the critical period, is sufficient to allow appropriate network tuning in these mutants, leading to the emergence of larvae whose seizure mutant phenotype has been completely rescued^5^. At the single cell level, excitatory synaptic currents exhibit a significantly extended duration after transient activity manipulations during the critical period, leading to increased excitatory synaptic drive indicative of change to the excitation:inhibition balance^5^.

Here we investigated how, during the critical period, neuronal activity mediates lasting network change. We identified Nitric Oxide (NO)-signaling as a downstream mediator of neuronal activity that is necessary for network adjustment during the critical period. We find that manipulation of NO-signaling is both sufficient and necessary to mimic or block the effects of activity perturbation during the critical period. Our results thus provide new mechanistic understanding for how activity, during a critical period, is transduced to alter key signaling properties of a mature circuit.

## Results

### Activity-manipulation during embryogenesis is sufficient to alter synaptic drive and network stability

We have previously shown that transient perturbation of network activity, during a critical period of nervous system development in late embryogenesis (17–19 h AEL), leads to lasting changes in both neuronal and network properties. Specifically, transient critical period activity perturbations cause broadening of excitatory cholinergic synaptic inputs (termed spontaneous rhythmic currents, SRCs) to identified motoneurons, manifest days later when recorded at the third instar stage (L3). This effect is accompanied by a significantly prolonged time needed to recover from electroshock-induced seizures, indicative that perturbing activity levels in a developing network is detrimental to post-embryonic network stability^5^. The same outcome is observed with different activity perturbations (optogenetic, genetic or chemical manipulations)^5^, as summarized in Figure S1.

**Figure 1:**
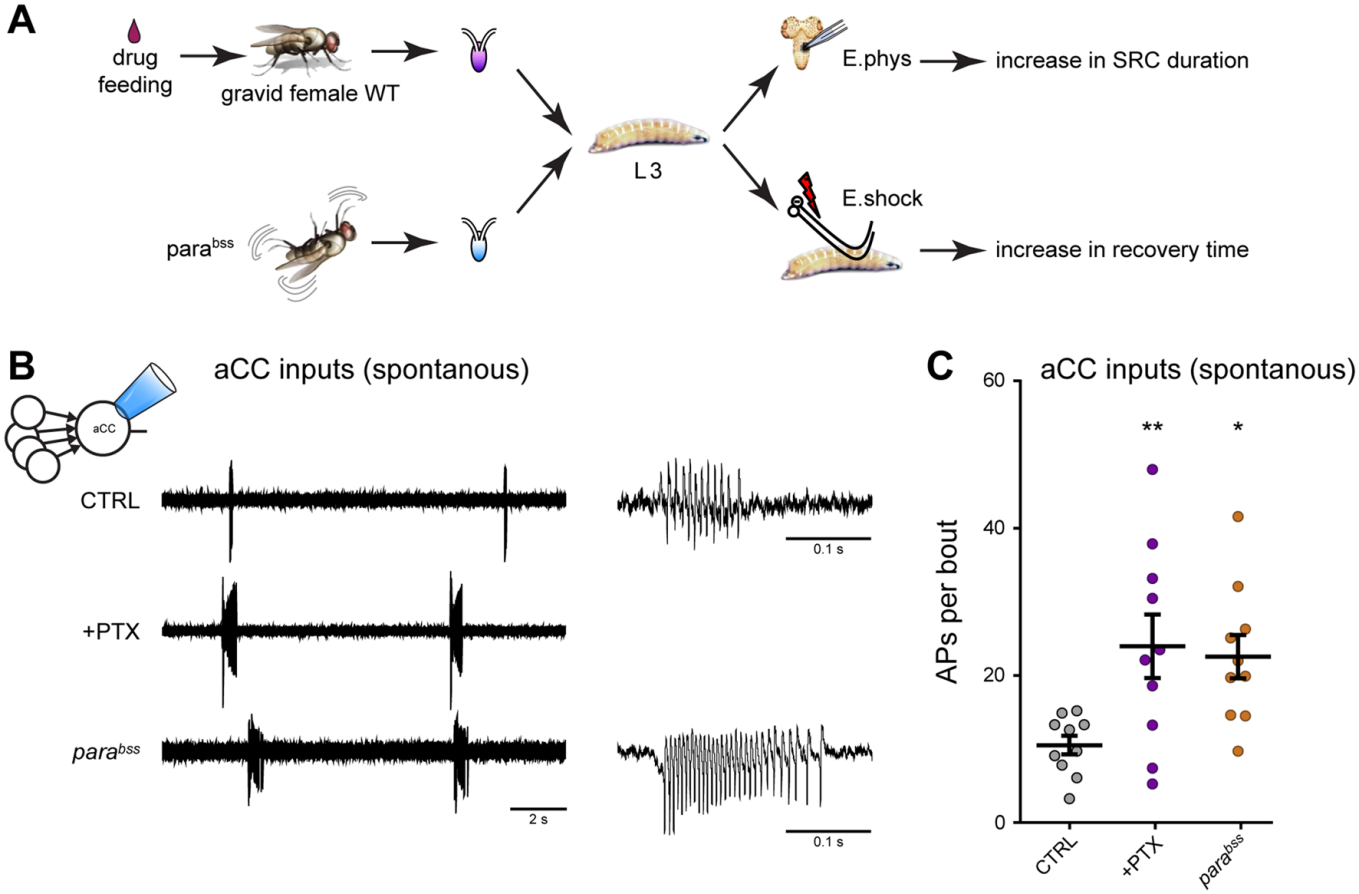
Activity manipulation during the embryonic critical period affects synaptic excitation of the aCC motoneuron. (**A**) Schematic representation of the experimental procedure. Early chemical manipulation was achieved by feeding PTX to wild-type gravid females. The seizure-prone *para*^*bss*^ mutant was also used to investigate hyper-excitability^48^. Five days later, wall-climbing L3 were tested by either electrophysiological recording from aCC motoneurons or by electroshock, in order to measure change to synaptic drive or susceptibility to induced seizure, respectively (see Figure S1). (**B**) Loose patch recordings from L3 aCC motoneurons showing increased endogenous (i.e. spontaneous) spiking activity in conditions of excessive excitation (PTX and *para*^*bss*^, see also Figure S1). (**C**) Quantification of the number of action potentials per bout. One-way ANOVA (F_(2, 27)_ = 3.91, *p* = 0.03) followed by Bonferroni’s *post-hoc* test (*n* = 10 in each group).

To show how increased duration SRCs influence motoneuron firing, we utilized loose-patch recordings that do not, unlike patch recording, disturb the intracellular compartment of neurons^9^. Working with the well characterized ‘aCC’ motoneuron, we recorded spontaneous activity bouts generated by the locomotor central pattern generator with rhythmic network activity^10^. We observed that critical period manipulations, sufficient to lead to increased SRC duration, also cause significantly increased bouts of action potential (AP) firing in this motoneuron. Thus, perturbing activity during the critical period with either embryonic exposure to the proconvulsant picrotoxin (a GABA_(A)_ receptor blocker, 23.97 ± 4.31 APs per bout, *p* = 0.0098), or by use of the *para*^*bss*^ background (22.55 ± 2.95, *p* = 0.0215; vs. CTRL: 10.53 ± 1.26, Fig. 1A–C), results in increase AP firing, consistent with increased excitation.

### Nitric oxide mimics the effect of activity-manipulation on seizure induction

**Figure 2:**
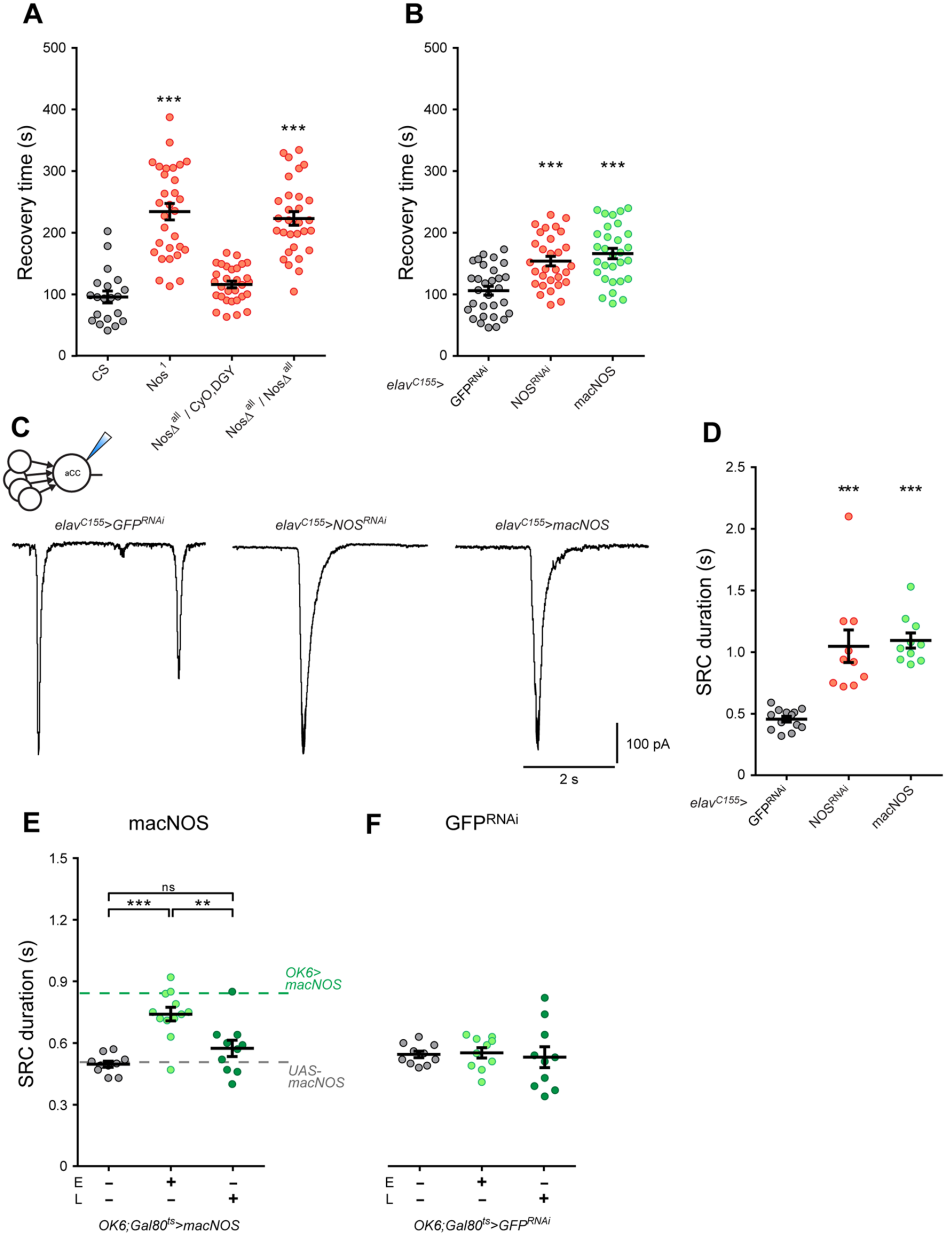
Manipulation of NOS is sufficient to mimic activity-manipulation in developing embryos. (**A**) Electroshock of L3 larvae showed a significant increase in RT in NOS mutants (Nos^1^ and NosΔ^all^). One-way ANOVA (F_(3, 106)_ = 44.73, *p* < 0.0001) followed by Bonferroni’s *post-hoc* test, *n* = 20 in control, *Canton S*, *n* = 30 in the other groups). The hemizygotic CRISPR mutant ((NosΔ^all^/CyO, DGY) exhibited no increase in RT: 116 ± 5 s, *p* = 0.6104). (**B**) Genetic downregulation of NOS (NOS^RNAi^) or overexpression of macNOS are each sufficient to increase RT (*elav*^*C155*^ > *NOS*^*RNAi*^: 154 ± 8 s, *elav*^*C155*^ > *macNOS*: 166 ± 8 s *vs.* CTRL: 101 ± 8 s). One-way ANOVA (F_(2,87)_ = 16.55, *p* < 0.001) followed by Bonferroni’s *post-hoc* test, ***p < 0.0001, *n* = 30 in each group. (**C**) Whole-cell recordings of endogenous SRCs from L3 aCC following pan-neuronal (*elav*^*C155*^) genetic manipulation of NOS. (**D**) Knock-down (NOS^RNAi^, *n* = 10) or overexpression of macNOS (*n* = 10) significantly increased SRC duration. GFP^RNAi^ was used as control (*n* = 13). One-way ANOVA (F_(2, 30)_ = 22.43, *p* < 0.0001) followed by Bonferroni’s *post-hoc* test, ***p < 0.0001. (**E**) Temporal regulation of macNOS expression in motoneurons (OK6 > Gal$) was achieved through GAL80^ts^ (“ + ” or “ − ” shows macNOS expression to be activated or suppressed, respectively). GAL4-mediated expression of macNOS during embryogenesis, but not during larval stage, led to an increase in synaptic current duration at L3. One-way ANOVA (F_(3, 29)_ = 16.36, *p* < 0.001) followed by Bonferroni’s *post-hoc* test, ***p < 0.001, ***p = 0.0021. Dotted lines represent reference values obtained from *OK6* > *macNOS* and, as a further control, the *UAS-macNOS* parental line. (**F**) Under identical temperature-controlled conditions, the expression of GFP^RNAi^, used as an additional control, did not show detectable change in synaptic current duration (*n* = 10 in each group).

To identify mechanisms underlying activity-perturbation during the critical period, we focused on nitric oxide (NO)-signaling, since this has been shown sufficient to alter synaptic drive and is regulated by activity^11^. First, we tested two well-characterized loss-of-function alleles of the single nitric oxide synthase (NOS) gene in *Drosophila*: *Nos*^*1*^ and NosΔ^all^^12,13^. Electroshock of both mutants (a rapid diagnostic for network instability), at L3, showed an expected increase in recovery time (RT) (Nos^1^, 234 ± 13 s, and NosΔ^all^, 223 ± 11 s, vs. *Canton S*: 95 ± 9 s, *p* < 0.0001, Fig. 2A). Pan-neuronal (*elav*^*C155*^*-Gal4*) knock-down of NOS, or over-expression of a constitutively active *NOS* transgene, *macNOS*^13^ were equally sufficient to induce increased seizure duration (Fig. 2B). These results indicate that bidirectional changes in NO-signaling produce an unstable network prone to seizure, thus mirroring our previous observations with activity manipulations^5^.

To confirm that manipulation of NO-signaling also influenced SRCs, we expressed either *NOS*^*RNAi*^ or the constitutively active *macNOS* in all neurons (Fig. 2C,D). Both increased SRC duration in L3 aCC (*NOS*^*RNAi*^: 1.05 ± 0.13 s, *p* < 0.0001; *macNOS*: 1.09 ± 0.06 s, *p* < 0.0001 *vs*. the control *GFP*^*RNAi*^: 0.45 ± 0.02 s). A caveat to this genetic manipulation is that the Gal4 driver line used expresses throughout both embryonic and larval periods. To demonstrate that embryonic manipulation of NO-signaling is sufficient to alter synaptic drive, we combined a temperature sensitive Gal4 inhibitor, Gal80^ts^ with the motoneuron driver OK6-Gal4. Restricting expression of macNOS to motoneurons during embryogenesis (*OK6;Gal80*^*ts*^ > *macNOS*) resulted in a significant increase in SRC duration in aCC recorded in L3 (from 0.50 ± 0.01 s to 0.74 ± 0.03 s, *n* = 12, *p* < 0.001, Fig. 2E). By contrast, no change was detected when expression of macNOS was restricted to postembryonic larval stages (0.57 ± 0.04 s, *n* = 10, *p* = 0.31). The same temperature shifts, repeated using a control genotype (*OK6;Gal80*^*ts*^ > *GFP*^*RNAi*^), showed no significant effects (Fig. 2F). This is consistent with NO-signaling being important during the critical period of network development.

### Nitric oxide is necessary for activity-dependent manipulation during an embryonic critical period

**Figure 3:**
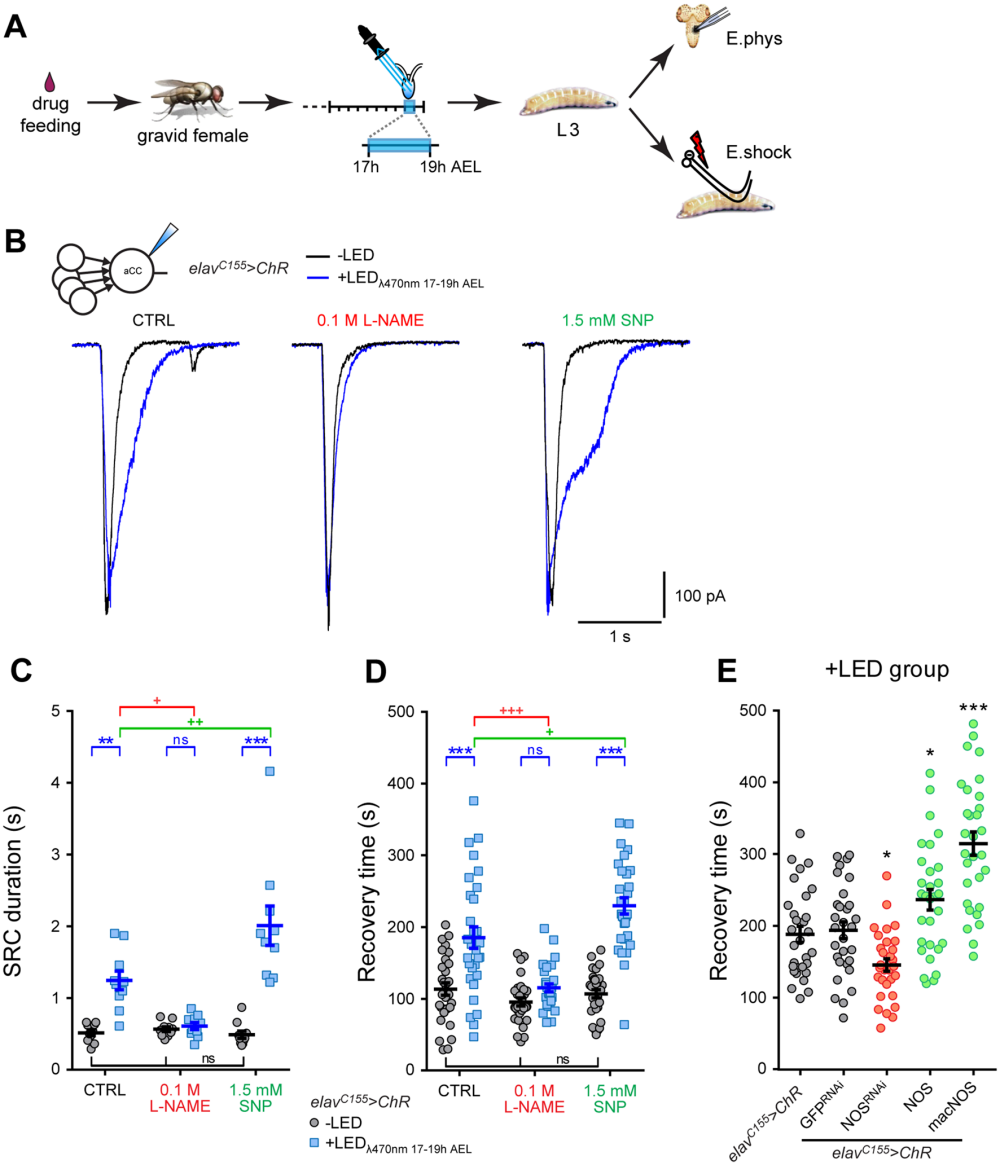
Nitric oxide mediates activity perturbation during the critical period. (**A**) Schematic representation of the experimental procedure. Early exposure to NOS drugs were achieved by feeding gravid females. We manipulated neuronal activity in embryos pan-neuronally expressing ChR by exposure to light between 17 and 19 h AEL (blue bar) which spans the identified critical period^5^. The resulting L3 were tested by either electrophysiological recording from aCC motoneurons or by electroshock. (**B**) Voltage-clamp recordings from L3 aCC show an increase in SRC duration following pan-neuronal activation of ChR during the critical period (*elav*^*C115*^ > *ChR*: + LED_λ470nm_ 17–19 h AEL, blue trace, *vs*. -LED, black trace). Inhibiting NOS (0.1 M L-NAME, see Figure S2A), prior to optogenetic manipulation, blocks this effect, while exposure to the NO donor (1.5 mM SNP, see Figure S2B) potentiates. (**C**) Quantitative analysis of SRC duration, *n* = 10 in each group. A two-way ANOVA shows significance for the + LED treatment (F_(1, 54)_ = 52.63, *p* < 0.001), NOS manipulation (F_(2, 54)_ = 13.16, *p* < 0.001), and interaction (F_(2, 54)_ = 16.4, *p* < 0.001). (**D**) RT to electroshock measured at L3 following the same stimulation protocol. Exposure to L-NAME blocked the ChR-induced increase in RT, while the NO donor SNP potentiated this effect, *n* = 30 in each group. A two-way ANOVA shows significance for the + LED treatment (F_(1, 174)_ = 86.43, *p* < 0.001), NOS manipulation (F_(2, 174)_ = 23.54, *p* < 0.001), and interaction (F_(2, 174)_ = 15.1, *p* < 0.001). **p < 0.01 and ***p < 0.001 are significant to + LED *vs*. -LED within each group. ^+^*p* < 0.05, ^++^*p* < 0.01 and ^+++^*p* < 0.001 show significance between NOS drugs and CTRL (+ LED groups *vs*. CTRL + LED group), Bonferroni’s *post-hoc* test. (**E**) RT from electroshock of L3 pan-neuronally co-expressing various transgenes together with ChR. Embryos were exposed to light during the critical period (+ LED group: λ470 nm, 100 ms, 17–19 h AEL). NOS inhibition (NOS^RNAi^) reduced the expected ChR-induced increase in RT. By contrast, up-regulation of NOS-signalling (NOS and macNOS) potentiated the effect of ChR activation compared to control (elav^C155^ > ChR). Co-expression of GFP^RNAi^, an additional control, showed no effect. One-way ANOVA (F_(4, 145)_ = 25.38, *p* < 0.001) followed by Bonferroni’s *post-hoc* test, *n* = 30 in each group, *p = 0.0312 and 0.0324, respectively, ***p < 0.001.

Because the phenotype of NO-signaling manipulations during the critical period mimics that of neuronal activity perturbations, we asked if the effects that neuronal activity exerts onto the developing network were mediated by NO-signaling. If so, then one would expect that the effects of neuronal over-activation might be rendered ineffective through concomitant reductions of NO-signaling and vice versa. To test this, we carried out such an interaction experiment: we increased network activity, only during the critical period, by pan-neuronal activation of channelrhodopsin (*elav*^*C155*^ > *ChR, 17–19h AEL*), while simultaneously altering NO-signaling through pharmacological manipulation. ChR is a blue-light sensitive, anionic channel, that results in neuron depolarization when activated. We had previously shown that drugs fed to gravid females enter the embryo and affect the critical period of nervous system development, but are subsequently efficiently metabolized and excreted, such that they are no longer detectable in late larval stages^14^. Feeding gravid females sufficient amounts of compounds known to either reduce NO-signaling (N(G)-nitro-L-arginine methyl ester (L-NAME) 0.5 M) or, conversely, increase NO levels (sodium nitroprusside (SNP) 5 mM), cause comparable seizure phenotypes as genetic manipulations that similarly change NO-signaling (cf. Fig. 2B). At lower concentrations (for L-NAME = 0.1 M, for SNP = 1.5 mM), however, these compounds do not affect recovery times following electroshock (Figure S2A-B). We then asked how exposure of embryos to these sub-threshold levels might modify responses to optogenetic activity manipulations during the critical period. Recordings from aCC motoneurons in control L3 larvae showed that optogenetic overactivation alone (i.e. in the absence of NO manipulation, water vehicle only) caused the expected increase in SRC duration (0.52 ± 0.04 *vs*. 1.25 ± 0.13 s, -LED vs. + LED, respectively, *p* = 0.003, Fig. 3A–C). However, SRC broadening was abolished following embryonic exposure to a sub-threshold dose of the NOS inhibitor, L-NAME (0.1 M, 0.57 ± 0.03 vs. 0.61 ± 0.05 s, − LED vs. + LED, respectively, *p* > 0.9). Conversely, the effects on SRCs were potentiated by exposure to a sub-threshold dose of the NO-donor, SNP (1.5 mM, 0.49 ± 0.05 to 2.01 ± 0.27 s, − LED vs. + LED, respectively, *p* < 0.001).

Embryonic exposure to L-NAME similarly suppressed electroshock-induced seizures that otherwise results from pan-neuronal optogenetic critical period manipulation (0.1 M L-NAME: 96 ± 6 vs. 115 ± 6 s, − LED vs. + LED, respectively, *p* > 0.9; water: 113 ± 9 vs. 185 ± 15 s, -LED vs. + LED, respectively, *p* < 0.0001, Fig. 3D). This response was, on the other hand, significantly enhanced by embryonic exposure to SNP (107 ± 6 s vs. 230 ± 11 s, *p* < 0.001). We tested additional drugs and consistently found that inhibitors of NO-signaling suppressed increases in seizure recovery time normally caused by critical period hypeactivity, whilst NO-activators enhanced the effect (Figure S3A).

To complement pharmacological manipulations, we repeated experiments coupling optogenetics to NO-signaling genetic manipulations. Mirroring the effect of the NOS-inhibitor, L-NAME, pan-neuronal expression of *NOS*^*RNAi*^ was sufficient to suppress the increase in seizure normally produced by optogenetic network activation during the critical period (*elav*^*C155*^ > *ChR;NOS*^*RNAi*^: 145 ± 9 s, *p* = 0.0312). Conversely, expression of *Drosophila NOS* resulted in a potentiation, mimicking the effect of the NO donor, SNP (*elav*^*C155*^ > *ChR;NOS*: 237 ± 14 s, *p* = 0.0324, Fig. 3E). As might be expected, expression of a constitutively active *macNOS,* to maximally increase NO-signaling^13^, resulted in an even stronger potentiation of seizure (*elav*^*C155*^ > *ChR;macNOS*: 315 ± 16 s, *p* < 0.001), indicative of a dose–response.

To further test the model of NO-signaling acting as an effector pathway downstream of neuronal activity, we tested its interaction with artificially decreased network activity. We have previously shown that both increasing as well as decreasing network activity, during the critical period, leads to similar phenotypes: broader SRCs and increased recovery times from electroshock-induced seizures^5^. If our model is correct, then pharmacological modulators of NO-signaling should have the opposite effects to SRC and seizure recovery phenotypes in the context of decreasing vs. increasing network activity during the critical period. Indeed, this is what we find when repeating pharmacological manipulation of NOS in the context of embryonic activity reduction via pan-neuronally expressed halorhodopsin (an orange-light sensitive chloride pump that hyperpolarizes neurons when active, Figure S3B).

### NO-signaling regulates network synaptic strength, affecting motoneuron inputs

**Figure 4:**
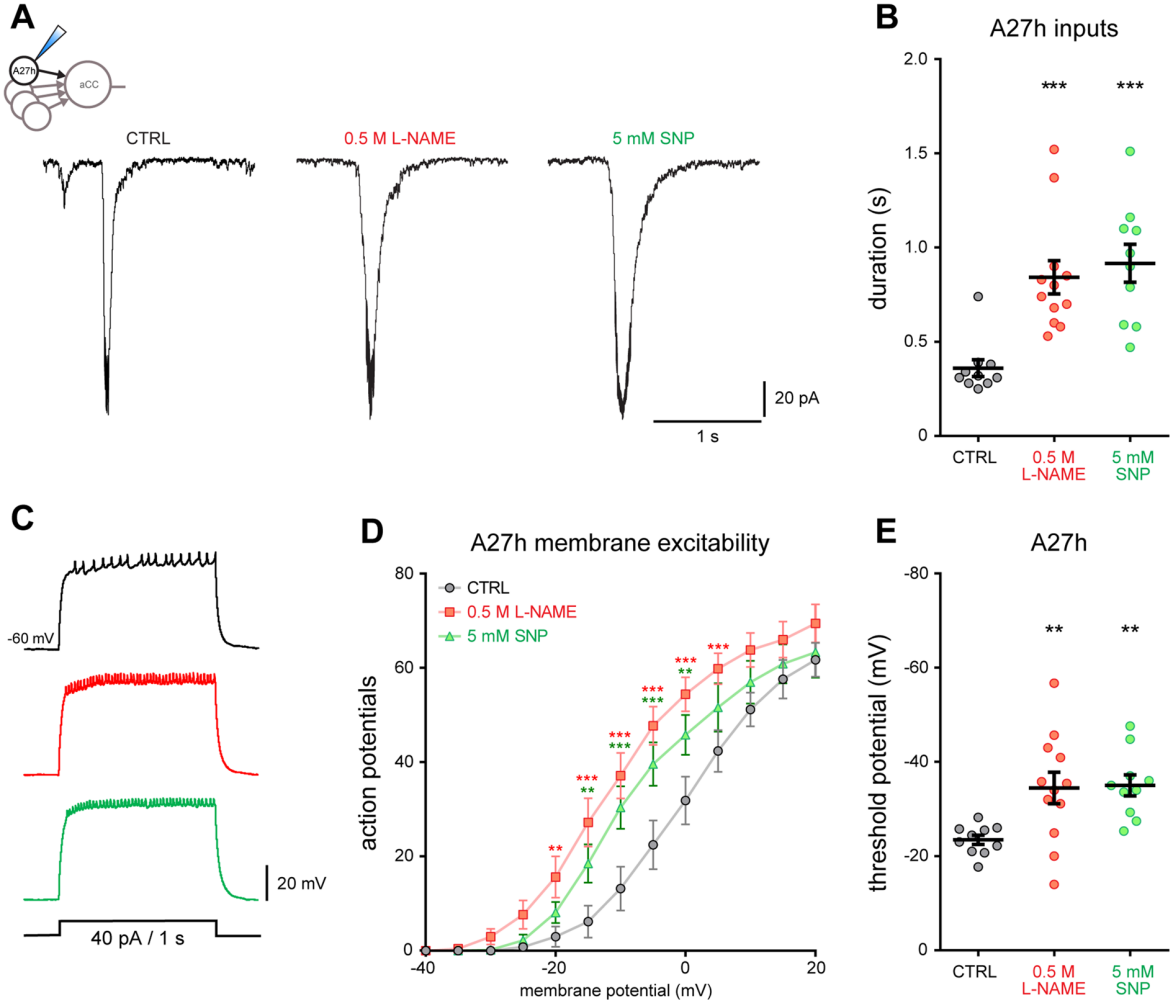
Manipulation of NOS modulates synaptic drive and excitability of the premotor interneuron A27h. (**A**) Voltage-clamp recordings of synaptic currents from the premotor interneuron A27h at L3 following NOS manipulation. Early administration of both 0.5 M L-NAME (*n* = 12) or 5 mM SNP (*n* = 10) increased duration of A27h synaptic currents (*n* = 10). (**B**) Quantification of the duration of A27h synaptic inputs. One-way ANOVA (F_(2, 29)_ = 12.72, *p* < 0.001) followed by Bonferroni’s *post-hoc* test. (**C**) Representative traces showing the effect of NOS manipulation on AP firing recorded from A27h and elicited by a depolarizing current injection (40 pA/1 s). (**D**) Changes in membrane excitability of L3 A27h interneurons were determined by injection of successively greater depolarizing current pulses (Δ + 4 pA steps/1 s). A two-way ANOVA revealed a significant effect of voltage (F_(13, 377)_ = 197.1, *p* < 0.001), NOS manipulation (F_(2,29)_ = 7.005, *p* = 0.003), and interaction (F_(26, 377)_ = 5.626, *p* < 0.001). Data are represented as mean ± SEM. **p < 0.01 and ***p < 0.001, Bonferroni’s *post-hoc* test, *n* = 10 in CTRL and 5 mM SNP, *n* = 12 in 0.5 M L-NAME. (**E**) NOS manipulation shifted the threshold potential in A27h interneurons toward more negative values. One-way ANOVA (F_(2, 29)_ = 6.306, *p* = 0.0053) followed by Bonferroni’s *post-hoc* test.

We sought to better understand the mechanistic basis for enhanced synaptic drive to aCC motoneurons, caused by transient network activity manipulations during the embryonic critical period. We investigated if the characteristic broadening of SRCs might be due to increased and/or prolonged presynaptic activity. To do so, we focused on one of the major cholinergic premotor interneurons, called ‘A27h’, which synaptically excites aCC motoneurons^15^, and made recordings in larvae that had experienced embryonic manipulation of NOS, by exposure to either L-NAME (0.5 M) or SNP (5 mM) at supra-threshold doses that reliably induce SRC broadening and increased recovery times from induced seizures (Figure S2A-B). We find that these embryonic NOS manipulations caused an increase in duration of synaptic currents recorded in A27h at L3 (0.84 ± 0.09 s, *p* = 0.0005, and 0.92 ± 0.10 s, *p* = 0.0002, respectively; CTRL: 0.36 ± 0.04 s, Fig. 4A,B). Membrane excitability of A27h also increased following manipulation of NOS activity during embryogenesis (Fig. 4C,D). In line with this result, we further observed that exposure to either L-NAME (0.5 M) or SNP (5 mM) shifted the threshold potential for observed spiking to more negative values (− 23.45 ± 0.99 mV, CTRL, *n* = 10 vs. − 34.46 ± 3.34 mV L-NAME, 0.5 M, *n* = 12, *p* = 0.0088 vs. − 35.00 ± 2.23 mV SNP, 5 mM, *n* = 10, *p* = 0.0084, Fig. 4E). The changes recorded from the A27h pre-motor interneuron are similar to those recorded from aCC motoneurons following critical period manipulations. This suggests that transient, embryonic manipulation of NO-signaling, within the developing network, causes a lasting change in synaptic strength across multiple components, affecting the synaptic drive to both motor-and pre-motor interneurons by altering intrinsic excitability and synaptic excitation.

## Discussion

Activity perturbation during a developmental critical period can induce permanent change to neural circuit function^3,4,16^. However, the underlying mechanisms that transduce this, and how affected networks respond to such changes, is not understood. In part, this is because knowledge of critical periods has been largely derived from complex mammalian neural circuits (e.g. vision) that deter single cell resolution. In this respect, it is significant that we previously identified a critical period during the development of the *Drosophila* larval locomotor circuitry, which seems to be analogous to mammalian critical periods in that transient activity perturbations during this developmental phase have a lasting effect, leaving the mature network in an altered state that is more susceptible to induced seizures^5^. Currently, we have limited understanding about the cellular or molecular mechanism by which activity, during a critical period, directs subsequent network development. By being able to work with identified neurons in a comparatively simple and extensively characterized network we have been able to identify NO as a key signaling component downstream of neuronal activity during this phase of network development.

We observed that network activity perturbations, whether increased excitation or inhibition, lead to similar lasting changes, namely of increased neuronal excitability and extended synaptic current duration that we recorded both in the aCC motoneuron, and also one of its main presynaptic excitatory pre-motor interneurons, A27h. At the level of the network, the impact of these and potentially other changes is that animals need a significantly prolonged time to recover from electroshock induced seizures. These synaptic, cellular and network/behavioural phenotypes mimic precisely those present in accepted *Drosophila* models of human epilepsy: the so-called bang-sensitive mutations, which we had previously characterized. Motoneurons in the bang-sensitive mutants also show synaptic currents with extended duration, and animals have a prolonged recovery from electroshock-induced seizures^14,17^. This tight correlation allows us to utilize the behavioral response to electroshock as a proxy for normal network development. Similar epilepsy syndromes exist in mammals where exposure to stressors, loud sound, flashing lights or electroshock, is equally sufficient to induce a seizure state^18–20^. That seizure occurs is indicative of underlying networks lacking robustness and which are unable to compensate for extremes of activity. At least part of the lack of robustness, in *Drosophila* seizure mutants, may be due to changes in phase relationships of network activity, as evidenced by abnormally increased synchrony of motoneuron activity between segments^21^. Increased synchrony within neuron subpopulations is also a hallmark of mammalian epilepsy^22–24^.

How transient activity manipulations during a critical period cause lasting changes to network properties, including increased likelihood of inappropriate synchrony and reduced robustness to activity challenges, remains to be determined. An experimental model system such as the *Drosophila* locomotor network can be uniquely helpful in this endeavor; it allows working with identified cells of known properties and connectivity. Our results show that NO-signaling mediates specification of both neuronal excitable and synaptic transmission properties, in at least two layers of the locomotor network. This is in line with increasing evidence for nitrergic modulation of rhythmic motor activity produced by central pattern generators in both vertebrates^25–27^ and invertebrates^28,29^. It is conceivable that change to synaptic transmission results from structural change to synaptic architecture in either, or both, pre and postsynaptic compartments. NO-signaling has been reported to alter synaptic structure^30^. Equally, change to presynaptic release, or changes to postsynaptic receptive field and/or membrane excitability in the postsynaptic motoneuron, may contribute. With respect to membrane excitability, NO has been reported to increase intrinsic excitability in motoneurons, for example, by inhibition of TASK-like K^+^ leak channels^31^.

Network tuning requires constituent neurons to be able to “sense” not only their own activity but, importantly, the activity of other neurons within the circuit, regardless of whether they are directly connected or not. NO, acting as a diffusible signal, is an obvious candidate to fulfil this important role^32–35^. Indeed, NO has been postulated to act as a volume transmitter synchronizing activity across neuron populations^36^. Our results support this hypothesis. It is notable that, in this context, that both increasing or decreasing NO-signaling generates the same outcome. This mirrors what we previously described (and repeated here) for relative activity levels during the critical period. Importantly, when testing the relationship between neuronal activity and NO-signaling, this provided evidence of a clear link between neuronal activity simulating NOS activity. That increasing or decreasing NO levels (or neuronal activity) have similar effects in destabilizing a developing network may suggest that network development is reliant on a ‘physiologically-appropriate’ activity balance and, moreover, that a shift away from this (positive or negative) is sufficient to induce permeant change to network dynamics. Such a reliance may explain why wildtype *Drosophila* larvae fed with phenytoin exhibit a significantly longer recovery time to electroshock^14^, an effect also observed in wildtype rats^37,38^. The presence of phenytoin, in an otherwise normal CNS, might be expected to alter activity away from a ‘norm’ that a developing network is expecting, and indeed, requires to set appropriate homeostatic set-points that remain fixed thereafter^39^.

*Drosophila* expresses one variant of nitric oxide synthase (NOS), but alternative splicing may further increase variability in protein function. The amino acid sequence shows ~ 40% overall sequence identity to mammalian inducible NOS, with the central region increasing to 61% identity to rat inducible NOS^40^. Immunohistochemistry shows that dNOS is first expressed in embryos, at stage 15^41^, which is some hours prior to the critical period. Our data indicate that change in NO levels during the critical period, stimulated by neuronal activity, rather than the absolute direction of change, seems the key determinant that is crucial for network tuning. This reveals a fundamental feature of network tuning during the critical period of development, namely of a non-linear system with an optimal state, which endows appropriate phase relationships needed for normal behavioral output as well as maximal robustness. The molecular mechanisms by which transient critical period manipulations cause lasting impact, remain to be determined, though our previous and current investigations point to changes in neuronal setpoint specification^5^. From a circuit perspective in particular, the establishment of a tractable experimental model system, which has an explicit mammalian-like critical period, has significant potential to greatly facilitate understanding of these enigmatic periods in neural circuit development.

## Materials and methods

### *Drosophila* rearing and stocks

All *Drosophila melanogaster* strains were grown and maintained on standard corn meal medium at 25 °C under a 12:12 h light–dark schedule. Fly stocks include the wildtype strain, Canton S (obtained from Bloomington Drosophila Stock Center), and the following previously described transgenic stocks: *UAS-GFP*^*RNAi*^ (#9331, BDSC), *UAS-NOS*^*RNAi*^ (#50675, BDSC), *UAS-NOS* (#56830, BDSC), *UAS-macNOS* and Nos^Δall^ (a gift of Prof. Oren Schuldiner), *Nos*^*1*^*/CyO, act* > *GFP* (#56822, BDSC).

To optogenetically manipulate neurons we used the following lines: channelrhodopsin (Nagel et al., 2005) – *UAS-ChR2*_*H134R*_*;cry*^*03*^ (a gift of Dr. Stefan Pulver) and halorhodopsin (Inada et al., 2011) – *UAS-eNpHR::YFP-50C;UAS-eNpHR::YFP-19C, UAS-eNpHR::YFP-34B* (a gift of Prof. Akinao Nose).

Expression driver lines include: *elav*^*C155*^*-Gal4* – for pan-neuronal expression, *elav*^*C155*^*-Gal4;cry*^*03*^ – for experiments involving embryonic exposure to blue light, in order to avoid activation of cryptochrome-expressing neurons^42–44^. *OK6-Gal4* – for expression in motoneurons. Temporal control of expression was achieved with the line *Gal80*^*ts*^*;OK6-Gal4*, which has been created by crossing *tub* > *Gal80*^*ts*^ and *OK6-Gal4* (both obtained from Bloomington Drosophila Stock Center). *A27h-Gal4* (*R36G02-Gal4*) – for expression in the premotor interneuron A27h^15^. This line was crossed with *UAS-mCD8::GFP* (#5130, BDSC) to facilitate visual identification of A27h during electrophysiological recordings.

### Drug treatments

Embryonic exposure to Picrotoxin (PTX, Sigma-Aldrich) was achieved by feeding gravid females to yeast extract supplemented with 0.25 mg/ml PTX for three days, prior to embryo collection. Embryos were collected as previously described and transferred to nondrug-containing vials.

Chemical manipulation of the NO pathway was performed using the same basic drug-feeding procedure. From our preliminary experiments, drug concentration was revealed as critical. Feeding gravid females to low doses of L-NAME (0.1 M, Sigma-Aldrich), inhibitor of NOS, was sufficient to affect the outcome of the optogenetic stimulation (increased RT and prolonged SRC duration) leaving the control group (-LED) unaltered. Conversely, higher doses (0.5 M), although more effective, were promoting the same features in absence of optical stimulation, affecting the control levels. We obtained identical results by testing other inhibitors/activators targeting the NO pathway or by overexpressing constructs such as NOS^RNAi^ or macNOS. Hence, for all the experiments shown in this paper, we first pre-determined an optimal concentration for each compound by testing a wide range of doses. Optimal concentrations of the drugs used are as follows: 5 mM 7-Nitroindazole (7-Ni), 10 mM Diethylenetriamine (DETA), 1.6 mM 1H-[1,2,4]oxadiazolo[4,3,-a]quinoxalin-1-one (ODQ), 0.5 mM Protoporphyrin IX (PPIX), 1.5 µM Rp-isomer guanosine 3',5'-cyclic monophosphate (RPcGMPs), 50 µM 8-Bromoguanosine 3′,5′-cyclic monophosphate (8-BR-cGMP)0.7-Ni, ODQ and PPIX were dissolved in 6% DMSO, therefore vehicle-treated embryos were also tested as a mock control. None of these concentrations were significantly affecting the control group (-LED), therefore data were presented as the percentage fold change between the experimental + LED and − LED group, normalised to a reference control group (water), by applying this formula: (‘experimental + LED/-LED fold change’) − (‘average of control + LED/− LED fold change’)/(‘average of control + LED/− LED fold change’). In this way, the average of all the control fold changes is zero; any increase is a positive value, any decrease is a negative value. RPcGMPs was purchased from Enzo Life Sciences (Exeter, UK); all remaining chemicals were obtained from Sigma-Aldrich (UK).

### Optogenetic manipulation of neuronal activity

Mated adult females were allowed to lay eggs on grape agar (Dutscher, Essex, UK) plates at 25 °C supplemented with a small amount of yeast extract paste (Melford). To ensure that embryos received enough retinal, adults were fed with 4 mM *all-trans*-retinal (Sigma-Aldrich, Poole, UK) dissolved in the yeast paste twice a day for three days prior to collection. Embryos were collected within a 4 h time range (time 0 ± 2 h after egg laying) and then transferred to a fresh grape agar plate. Plates were placed in a humidified atmosphere inside a 25 °C incubator and exposed to collimated light from an overhead LED, positioned to a distance of 17 cm from the embryos. LEDs had peak emission at λ470 nm (bandwidth 25 nm, irradiance 466 ± 14 nW cm^−2^; OptoLED, Cairn Instruments, Kent, UK) or λ565 nm (bandwidth 80 nm, 250 ± 10 μW cm^−2^; M565L2, Thorlabs, Newton, NJ) to activate ChR or eNpHR, respectively. Embryonic exposure to λ470 nm, but not λ565 nm is sufficient to induce a seizure-phenotype at L3^5^ through the activation of cryptochrome-expressing neurons^42–44^. Therefore, experiments involving blue light required a cry-null (*cry*^*03*^) background, to avoid unspecific effects.

Light was pulsed at 1 Hz using a Grass S48 stimulator (Grass instruments, Quincy, MA, USA). ChR was activated with short duration pulses (100 ms) while eNpHR required long light pulses (600 ms) in order to prevent rebound spike firing, as previously described^5^. Embryos were optically treated for a pre-determined time period during embryogenesis (between 17 and 19th ± 2 h AEL), which corresponds to the critical period^5^. After manipulation, embryos were transferred into food bottles and maintained at 25 °C in complete darkness until ~ 4 days later when wall-climbing L3 were collected and then tested electrophysiologically or behaviourally.

### Electrophysiology

Loose patch recordings were performed on L3 aCC motoneurons. Thin-wall borosilicate glass capillaries (GC100TF-10, Harvard Apparatus, Edenbridge, UK) were used to pull recording electrodes (unpolished) with resistances between 1.5 and 2.5 MΩ. Data were acquired with a sampling rate of 20 kHz, filtered with a low-pass filter of 0.2 kHz and analysed in Clampfit 10.4 (Molecular Devices, Sunnyvale, CA).

Whole cell voltage- and current-clamp recordings were achieved using thick-walled borosilicate glass electrodes (GC100F-10, Harvard Apparatus) fire polished to resistances of 10–15 MΩ (aCC) and 15–20 MΩ (A27h). Recordings were made using a Multiclamp 700B amplifier controlled by pCLAMP (version 10.4) via a Digidata 1440A analog-to-digital converter (Molecular Devices). Traces were sampled at 20 kHz and filtered online at 10 kHz. External saline composition was as follows: 135 mM NaCl, 5 mM KCl, 4 mM MgCl_2_·6H_2_O, 2 mM CaCl_2_·2H_2_O, 5 mM TES and 36 mM sucrose, pH 7.15. Current clamp recordings were performed in the presence of 1 mM mecamylamine to block endogenous cholinergic synaptic currents^45^. Internal patch solution was as follows: 140 mM K^+^-d-gluconate, 2 mM MgCl_2_·6H_2_O, 2 mM EGTA, 5 mM KCl, and 20 mM HEPES, pH 7.4. KCl, CaCl_2_, MgCl_2_ and sucrose were purchased from Fisher Scientific (Loughborough, UK); all remaining chemicals were obtained from Sigma-Aldrich (Poole, UK).

Spontaneous rhythmic currents (SRCs) were recorded from L3 aCC motoneurons for 3 min. Traces were sampled at 20 kHz and filtered at 0.2 kHz low pass. Cells with input resistance < 0.5 GΩ were not considered for analysis. Synaptic current parameters were examined for each recorded cell using Clampfit (version 10.4). To measure the amplitude of SRCs, the change from baseline to peak current amplitude was determined^46^. Currents shown were normalized for cell capacitance (determined by integrating the area under the capacity transient resulting from a step protocol from − 60 to − 90 mV). The duration of each synaptic event was defined as the time from current initiation until the return to baseline. Synaptic inputs to A27h were recorded with the same procedure. Selective overexpression of mCD8::GFP was used in all recordings performed from L3 A27h interneurons (by crossing the *A27h-Gal4* and *UAS-mCD8::GFP* lines), in order to facilitate visual identification.

Membrane excitability was determined as the number of APs evoked by a series of rectangular depolarizing current pulses (4 pA steps/1 s, from 0 to 100 pA). L3 A27h interneurons were recorded in current-clamp mode and held at − 60 mV before the start of the protocol. Recordings were performed in presence of 1 mM mecamylamine to block endogenous cholinergic synaptic inputs to A27h. The mean number of evoked action potentials elicited by incremental current injections was counted for each event. The input–output relationship was analysed using GraphPad Prism version 6 (GraphPad Software, San Diego, CA). Threshold potential in A27h was measured in current-clamp mode by slowly depolarising the cell until the first action potential was elicited.

### Electroshock assay

Electroshock assay was performed as previously described^14^. Briefly, wall-climbing L3 were transferred to a plastic dish after washing to remove food residue and gently dried using paper tissue. Once normal crawling behaviour resumed, a conductive probe, composed of two tungsten wires (0.1 mm diameter, ~ 1–2 mm apart) was positioned over the approximate position of the CNS, on the anterior-dorsal cuticle of the animal. A 2.3 V DC pulse for 2 s, created by a constant voltage generator (DS2A‐mkII, Digitimer Ltd., Welwyn Garden City, Hertfordshire, UK), was applied. In response to the electric stimulus, we observed a transitory paralysis status in which larvae were tonically contracted and, occasionally, exhibited spasms. The time to resumption of normal crawling behaviour was measured as recovery time (RT). Normal crawling was defined as a whole-body peristaltic wave resulting in forward movement.

### Quantification and statistical analysis

Data was acquired and imported into Microsoft Excel (Microsoft Corp., Redmond, WA). All statistical analyses were performed in GraphPad Prism (version 7). All data are presented as the mean ± SEM. This study includes electrophysiological recordings and electroshock assays of *Drosophila* third instar larvae. Sample size (*n*) was pre-calculated as follows. Pilot recordings of a small sample of cells (*n* = 5) analysed using Lenth’s power software^47^ indicated that 8 samples as being optimal for statistical power (*α* = 0.05 and power at 0.8). This is also in agreement with all prior publications from our group. Therefore, we decided to test 10 cells in 10 larvae (biological replicates) per group, unless stated otherwise in the text. Determination of statistical power for recovery times (RTs) from electroshock indicate that an *n* of at least 29 is sufficient (*α* = 0.05 and power at 0.8). To be consistent with our previous publication^5^, we used 30 larvae for each test. Each larva, mixture of both male and female, was considered as a biological replicate and was tested only once. Details including the exact value of *n* for each sample and *p* values are provided in the Results section or in each respective figure legend.

Statistical analyses were conducted using either unpaired *t*-test (in the text only), one-way, or two-way ANOVA as indicated in the respective figure legends. For one-way or two-way ANOVAs, a *post-hoc* Bonferroni multiple comparisons test was conducted. The null hypothesis was rejected at the 0.05 level. *p* values < 0.05 were considered significant. Significance was shown as * = *p* < 0.05, ** = *p* < 0.01, *** = *p* < 0.001, and not significant values were not noted or shown as ns. Figures were assembled with Adobe Illustrator CS3 (Adobe, San Jose, CA, USA).

## Supplementary information

Supplementary Information.

## Data Availability

All reagents described will be provided, if not covered by a material transfer agreement. All raw data is available on reasonable request.
