# Supplementary material for: Nitric oxide mediates activity-dependent change to synaptic excitation during a critical period in Drosophila
Source: Sci Rep. 2021 Oct 13;11:20286. doi: 10.1038/s41598-021-99868-8 (PMC8514485; doi:10.1038/s41598-021-99868-8)
Supplement: Supplementary file 1 — Supplementary Information. [file 41598_2021_99868_MOESM1_ESM.pdf]

Gicahello et al.,

Supplementary information

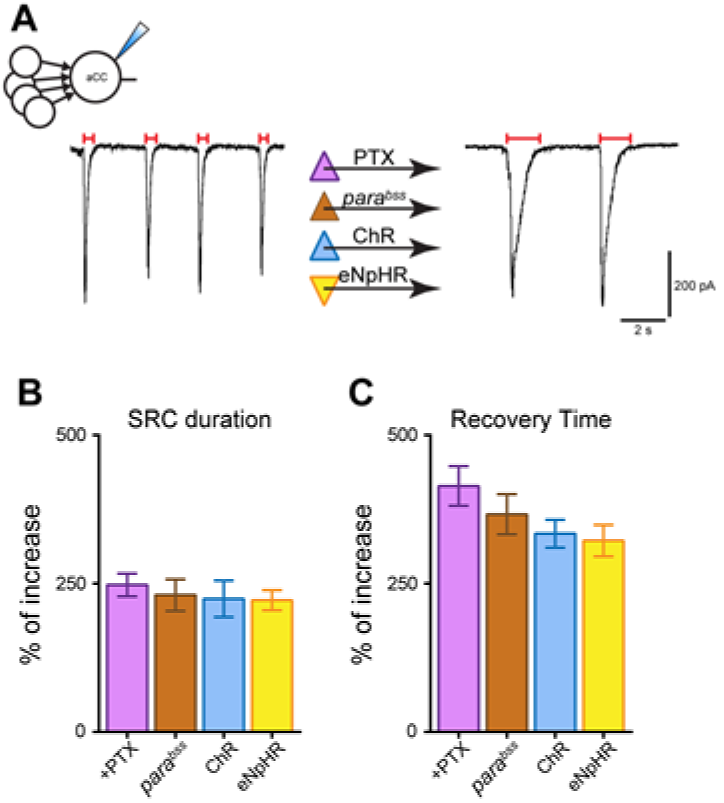

Figure S1. Activity manipulation during the embryonic critical period affects synaptic inputs to aCC motoneuron and induces a seizure phenotype.

(A-B) SRC broadening recorded from L3 aCC following activity manipulation during embryogenesis. Chemical (picrotixin, PTX), genetic (*para*^{bss}), or optogenetic manipulation (*ChR* or *eNpHR*) produces an identical increase in duration of SRCs recorded in the aCC motoneuron (B), which correlates with an increased recovery time from electroshock-induced seizure activity (C) (5).

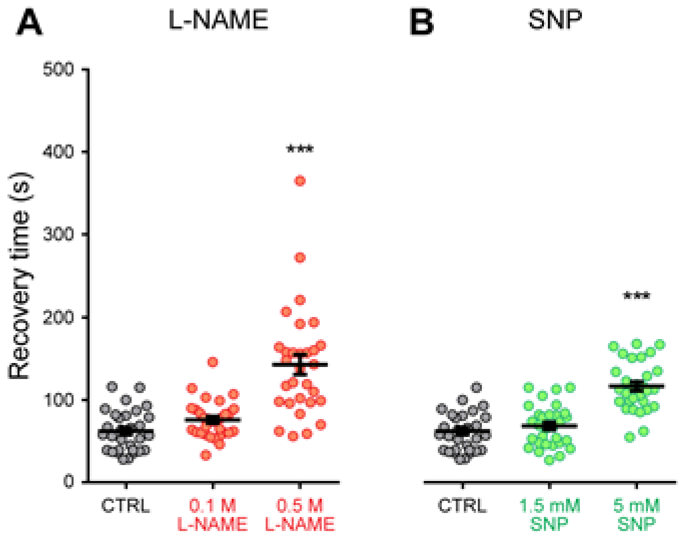

Figure S2. Higher doses of NOS drugs affect seizure induction, mirroring NOS genetic manipulation.

A) Embryonic exposure to 0.1 M L-NAME (NOS inhibitor), showed a similar RT to electroshock compared to the untreated control group (76 ± 4 *vs.* 62 ± 5 s, 0.1 M L-NAME *vs.* CTRL, $p = 0.6802$). Conversely, higher doses were sufficient to increase RT to electroshock (143 ± 12 *vs.* 62 ± 5 s, 0.5 M L-NAME *vs*. CTRL, ***$p < 0.0001$), mirroring NOS genetic manipulation. One-way ANOVA ($F_{(2, 87)} = 30.52$, $p < 0.0001$) followed by Bonferroni's post-hoc test, $n = 30$ in each group. B) Similarly, 1.5 mM SNP, NO donor, was not sufficient to produce an effect (68 ± 4 *vs.* 62 ± 5 s, 1.5 mM SNP *vs.* CTRL, $p = 0.99$), while 5 mM SNP significantly increased RT (116 ± 5 *vs.* 62 ± 5 s, 5 mM SNP *vs.* CTRL, ***$p < 0.0001$). One-way ANOVA ($F_{(2, 87)} = 36.69$, $p < 0.0001$) followed by Bonferroni's post-hoc test, $n = 30$ in each group.

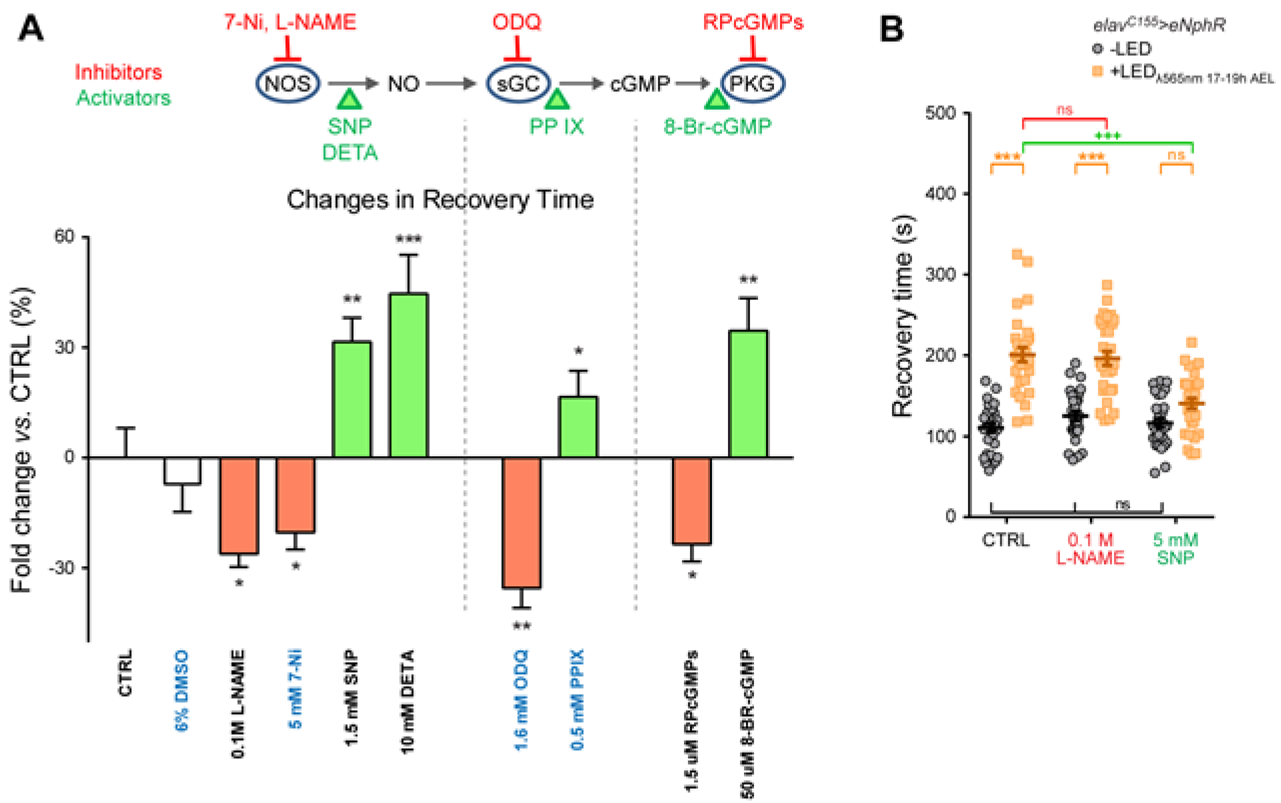

Figure S3. Nitric oxide mediates activity perturbation during the critical period.

(A) Upper shows schematic representation of the NO-signalling pathway. NOS: Nitric oxide synthase, sGC: soluble guanylyl cyclase, cGMP: cyclic guanosine monophosphate and PKG: Protein Kinase G. Inhibitors and activators were indicated in red and green, respectively. Chemical manipulation of the NO pathway (lower) affects the ChR-induced increase in RT to electroshock (*elav*^{C155}>*ChR*, LED_{100ms}, 17-19h AEL). Values are expressed as fold change (+LED/-LED) and normalised to control water (set to zero, see Materials and Methods). For each compound, drug concentration was first optimised to ensure no effect was observed in the -LED control group (One-way ANOVA $F_{(9, 290)}$ = 0.8452, *p* = 0.4987). Drugs labelled in blue were dissolved in 6% DMSO (which had no effect: -6.9 ± 7.9%) instead of water. All inhibitors significantly reduced the ChR-increase in RT (0.1 M L-NAME: -26.2 ± 3.6%, **p* = 0.016; 5 mM 7-Ni: -20.4 ± 4.6%,* *p* = 0.041; 1.6 mM ODQ: -36.53 ± 5.4%, ***p* = 0.005 and 1.5 µM RPcGMPs: -23.52 ± 4.7%, **p* = 0.018), while activators potentiated the effect of ChR activation (1.5 mM SNP: +31.5 ± 6.6%, ***p* = 0.0048; 10 mM DETA: +44.6 ± 10.6%, ****p* = 0.0002; 0.5 mM PPIX: +16.5 ± 7.1%, **p* = 0.0341 and 50 µM 8-BR-

cGMP: +34.5 ± 8.9%, ***p* = 0.0040). One-way ANOVA ($F_{(9, 290)}$ = 16.86, $p < 0.001$) followed by Bonferroni's *post-hoc* test, *n* = 30 in each group. (B) To test the contribution of NO-signalling in a context of neuronal inhibition, we repeated the pharmacological manipulation of NOS in embryos pan-neuronally expressing halorhodopsin (*elav^{C155}>eNpHR*, λ565 nm, 600 ms/1 Hz). L3 larvae from the CTRL group, lacking manipulation of NO-signalling, showed the expected increase in RT after electroshock (111 ± 5 *vs*. 201 ± 9 s, $p < 0.001$). Exposure to L-NAME (0.1 M, sufficient to inhibit the effect of ChR), did not prevent the effect of eNpHR-mediated inhibition (125 ± 5 *vs*. 196 ± 9 s, -LED *vs.* +LED, respectively, $p < 0.001$), exhibiting values statistically not different to those of the CTRL group (p > 0.9). Conversely, exposure to SNP (5 mM, sufficient to potentiate the effect of ChR) blocked the eNpHR-mediated increase in RT (116 ± 5 *vs*. 140 ± 6 s, -LED *vs*. +LED, respectively, p = 0.246). A two-way ANOVA analysis revealed a significant effect of the LED treatment ($F_{(1, 174)}$ = 118.7, $p < 0.001$), NOS manipulation ($F_{(2, 174)}$ = 12.66, $p < 0.001$), and interaction ($F_{(2, 174)}$ = 12.16, $p < 0.001$). ***$p < 0.001$ shows significance to +LED *vs*. -LED within each group; $^{+++}p < 0.001$ shows significance to NOS drugs (+LED groups *vs*. CTRL), Bonferroni's *post-hoc* test, *n* = 30 in each group.
